# Modelling prognostic factors in advanced pancreatic cancer

**DOI:** 10.1038/sj.bjc.6604568

**Published:** 2008-08-26

**Authors:** D D Stocken, A B Hassan, D G Altman, L J Billingham, S R Bramhall, P J Johnson, N Freemantle

**Affiliations:** 1 Cancer Research UK Clinical Trials Unit, University of Birmingham, Birmingham, UK; 2 Weatherall Institute of Molecular Medicine, University of Oxford, Oxford, UK; 3 Cancer Research UK Medical Statistics Group, Centre for Statistics in Medicine, University of Oxford, Oxford, UK; 4 Queen Elizabeth Hospital, Birmingham, UK; 5 Department of Primary Care and General Practice, University of Birmingham, Birmingham, UK

## Abstract

Pancreatic cancer is the fifth most common cause of cancer death. Identification of defined patient groups based on a prognostic index may improve the prediction of survival and selection of therapy. Many prognostic factors have been identified often based on retrospective, underpowered studies with unclear analyses. Data from 653 patients were analysed. Continuous variables are often simplified assuming a linear relationship with log hazard or introducing a step function (dichotomising). Misspecification may lead to inappropriate conclusions but has not been previously investigated in pancreatic cancer studies. Models based on standard assumptions were compared with a novel approach using nonlinear fractional polynomial (FP) transformations. The model based on FP-transformed covariates was most appropriate and confirmed five previously reported prognostic factors: albumin, CA19-9, alkaline phosphatase, LDH and metastases, and identified three additional factors not previously reported: WBC, AST and BUN. The effects of CA19-9, alkaline phosphatase, AST and BUN may go unrecognised due to simplistic assumptions made in statistical modelling. We advocate a multivariable approach that uses information contained within continuous variables appropriately. The functional form of the relationship between continuous covariates and survival should always be assessed. Our model should aid individual patient risk stratification and the design and analysis of future trials in pancreatic cancer.

Pancreatic ductal adenocarcinoma is a common cause of cancer death and is difficult to treat because clinical presentation is often late, and the disease is resistant to conventional chemotherapy. Long-term survival remains poor with a 5-year survival rate of 0.4–4% (Bramhall *et al*, 1995; Jemal *et al*, 2003). Multivariable prognostic models are important for grouping patients into risk sets for predicting survival and treating appropriately. There is currently no prognostic tool in routine use to identify subgroups of pancreatic cancer patients for selection and stratification of treatment and prediction of survival.

Because of its poor prognosis, few prognostic factors may be expected for patients with advanced pancreatic cancer; however, many possible factors have been identified. The majority of prognostic factor studies are questionable in terms of sample size and statistical methods, most based on small retrospective analyses. Literature searches (ISI Web of Science and Ovid Technologies databases) identified 36 prognostic factor studies reporting a total of 34 possible prognostic factors for advanced pancreatic cancer patients (Table 1) grouped as surgical, clinical, laboratory or demographic. Four studies (Johnson *et al*, 2001; Berlin *et al*, 2002; Ducreux *et al*, 2002; Maisey *et al*, 2002) were randomised controlled trials reporting five prognostic factors from multivariate analyses, namely metastases, tumour site, performance status, alkaline phosphatase and treatment. The remaining 32 studies (Friedman and van den Eeden, 1993; Yasue *et al*, 1994; Falconer *et al*, 1995; Lundin *et al*, 1995; Ishii *et al*, 1996; Rothenberg *et al*, 1996; Shibamoto *et al*, 1996; Cubiella *et al*, 1999; Storniolo *et al*, 1999; Halm *et al*, 2000; Terwee *et al*, 2000; Trigui *et al*, 2000; Ueno *et al*, 2000; Ikeda *et al*, 2001; Ridwelski *et al*, 2001; Tas *et al*, 2001; Tsuruta *et al*, 2001; Saad *et al*, 2002; Bachmann *et al*, 2003; Engelken *et al*, 2003; Fujino *et al*, 2003; Karayiannakis *et al*, 2003; Micke *et al*, 2003; Ohigashi *et al*, 2003; Paillaud *et al*, 2003; Stemmler *et al*, 2003; Talar-Wojnarowska *et al*, 2003; Ziske *et al*, 2003; Gupta *et al*, 2004; Kuhlmann *et al*, 2004; Watanabe *et al*, 2004; Ni *et al*, 2005) were based on consecutive series of patients, often retrospective, often single-centre, of which 15 studies were based on fewer than 100 patients (Yasue *et al*, 1994; Ishii *et al*, 1996; Rothenberg *et al*, 1996; Halm *et al*, 2000; Ikeda *et al*, 2001; Tsuruta *et al*, 2001; Saad *et al*, 2002; Karayiannakis *et al*, 2003; Micke *et al*, 2003; Ohigashi *et al*, 2003; Paillaud *et al*, 2003; Stemmler *et al*, 2003; Talar-Wojnarowska *et al*, 2003; Ziske *et al*, 2003; Gupta *et al*, 2004). One was based on five observational studies with varied inclusion criteria, inconsistent results and no prospective verification (Terwee *et al*, 2000). The largest series (2380 patients) identified factors based on univariate analyses and data containing a large proportion (57%) of censored patients (Storniolo *et al*, 1999).

An important issue in prognostic factor studies is the nature of the relationship between the factor and survival (functional form). Continuous variables are often simplified at analysis by assuming a linear relationship with log-hazard or by introducing a step function through categorisation (frequently dichotomisation). If the linearity assumption is not correct, the final prognostic model could be misspecified. Misspecification of the functional form may lead to inappropriate conclusions but has not been previously investigated in pancreatic cancer studies. Many researchers avoid this problem by dichotomising, with a consequent loss of power. There is also the risk of important bias when the choice of cutoff is data-driven and the use of different cutoff points across multiple studies hinders direct comparisons.

The aim of this study was to evaluate potentially important baseline prognostic factors for survival in advanced pancreatic cancer using prospective data from two randomised controlled trials and a total of 653 patients (Bramhall *et al*, 2001, 2002). The study investigated clinical, histological, biochemical and demographic variables. A multivariable approach was used accounting for the functional form of the relationship between continuous factors and survival. Models were developed either on the basis of standard assumptions of log linear or step functional relationships with survival, or a novel approach based on nonlinear relationships, using more complex fractional polynomial (FP) transformations: a flexible, parametric method for modelling nonlinear relationships (Royston and Altman, 1994; Altman and Lyman, 1998).

## PATIENTS AND METHODS

### Data

Two international phase III British Biotech studies (BB128, Bramhall *et al*, 2001; BB193, Bramhall *et al*, 2002) randomised 414 and 239 patients with advanced pancreatic cancer, respectively: BB128 randomised patients between marimistat and gemcitabine; BB193 randomised patients between marimistat with gemcitabine and gemcitabine alone. The studies had similar eligibility criteria: histologically or cytologically unresectable pancreatic cancer, within 8 weeks of diagnosis or disease recurrence and Karnofsky performance status of ⩾50% (BB128) or ⩾60% (BB193). Previous therapy for metastatic or locally advanced disease was an exclusion criterion. The primary outcome measure in both studies was survival time calculated from the date of randomisation to the date of death from any cause. Randomisation was stratified by cancer stage (stage I/II, III or IV), Karnofsky performance status (50–70%, 80–100%), sex and study centre. The first stage of data reduction was considering only factors that were clinically relevant and available within an NHS outpatient clinic. Eighteen baseline clinical, histological, biochemical and demographic variables (including trial and randomised treatment group) were considered appropriate for analysis as possible prognostic factors (Table 2).

### Statistical analysis

We followed a strategy aimed at maximising model performance and avoiding poorly fitted and overfitted regression models in the development (Harrell *et al*, 1996) and reporting (McShane *et al*, 2005) of multivariable prognostic models. Initial analysis was based on standard methodology comparing Kaplan–Meier survival estimates using the log-rank test and estimating univariate hazard ratios for levels of each factor. The hazard of death was assessed in the multivariable setting using Cox proportional hazards regression modelling with variable reduction by backward elimination. The proportional hazards assumption was investigated for each covariate using log cumulative hazard (Collett, 1994) and martingale residual plots, and incorporating a time-dependent covariate (*X*=factor(LN (survival)−LN (mean survival))) and did not indicate any significant violation.

Ten of the 18 possible prognostic factors were collected as continuous measurements. Continuous data were investigated by assessing three different assumptions of the underlying relationship between survival and predictor, they are: (a) a linear relationship between the predictor and log hazard, (b) a step functional relationship using dichotomised covariates (laboratory measures based on central laboratory reference ranges) and (c) a nonlinear relationships based on either a simple log or more complex nonlinear FP transformation (Royston and Altman, 1994). For the third model, the functional form of each variable was assessed univariately comparing the Akaike's Information Criterion (AIC) (Collett, 1994) of a model based on the simple log transformation with the AIC of a model based on the best fitting FP transformation. First- and second-degree FP transformations (Meier-Hirmer *et al*, 2003) were considered using a selection level of 0.05 for input of variables based on power values of the polynomial ranging (−2, −1, −0.5, 0 (log), 0.5, 1, 2, 3). The best FP for each predictor was selected if it resulted in a significantly better fit (significantly smaller AIC) than the log transformation. The most appropriate (log or FP) transformation, if any, was applied to each variable and all variables were considered multivariately using Cox proportional hazards regression based on a backward selection method using a nominal significance level of 0.05 for elimination and including trial, sex, cancer stage (stratification factors at randomisation) and randomised treatment in each model.

The majority of variables had ⩽5% missing values (Table 2). Tumour stage, CA19-9 and WBC had 5–10% missing values, and lymph node status was missing for 24% of patients. Metastases or lymph node status was considered in the analysis as dummy variables using ‘negative’ as a reference level. Primary analysis was based on complete cases and a secondary analysis used multiple imputation to investigate the possible influence of variables with larger amounts of missing data (Rubin, 1987; Schafer, 1997) and provided valid inferential alternative results.

Model fit was assessed comparing AIC statistics, deviance residuals and Kaplan–Meier survival statistics for four predictive groups. The four predictive groups were based on quartiles of linear predictor scores, assessed comparing median survival estimates and hazard ratios. The bootstrap resampling approach described by Harrell *et al* (1996) was applied to assess the extent of overfitting in the final model, using 200 bootstrap resamples. This approach repeats the model selection methods used in the original model development in a series of bootstrapped resamples, freezing the derived model and applying to the original sample. Model optimism (overfitting) is described by the difference in the rank correlation coefficient relating predicted and observed survival times between the model derived in the bootstrap resample and that from the frozen model applied to the original sample averaged over 200 resamples. This provides an honest estimate of internal validity penalised for overfitting (Harrell *et al*, 1996).

Analyses were carried out using SAS and R using a two-sided significance level of 0.05 throughout.

## RESULTS

### Patient characteristics

A total of 653 patients were randomised. The eighteen clinically appropriate factors for analysis are presented in Table 2 and appear balanced across the two studies. On average, patients in the two trials were randomised 20 and 15 days after diagnosis and started treatment the day following randomisation. The average age of patients was 63 years (range 29–89), 368 (56%) were male, 439 (68%) had cancer stage IV disease, 436 (67%) presenting with metastases and 251 (39%) had lymph node involvement.

### Survival

**Figure 1:**
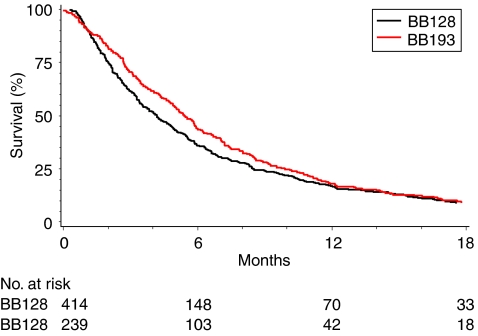
Survival function by trial.

The majority of patients (612, 94%) had died by the time of analysis with a median follow-up time of 21 months for the 41 patients still alive (Table 2, Figure 1). The median survival estimate for the group is 4.7 months (95% CI: 4.2, 5.1) with 12-month survival estimate of 17% (Figure 1). Hazard functions estimated for 1-monthly time intervals to 18 months from trial entry were similar for both trials and reasonably constant over time. No significant survival benefit for marimastat was identified in the BB128 trial (*P*=0.19) when compared with gemcitabine (Bramhall *et al*, 2001). Similarly, no significant survival benefit was seen for a combination of gemcitabine and marimistat when compared with gemcitabine alone in the BB193 trial (*P*=0.95) (Bramhall *et al*, 2002).

### Univariate analyses

Log-rank analyses (Table 3) indicated that potentially important factors were age (split at median, *P*=0.036), nodal status (*P*=0.035), cancer stage (I/II *vs* III/IV), metastases (both *P*<0.001) and laboratory measures (split as normal/abnormal according to laboratory reference ranges as per current clinical practise) as AST, alkaline phosphatase, albumin, LDH, WBC (all *P*<0.001), bilirubin (*P*=0.002), CA19-9 (*P*=0.005) and haemoglobin (*P*=0.009). Trial, treatment (gemcitabine *vs* marimistat), race (white *vs* not-white), sex, tumour stage (T0, 1, 2 *vs* T3, 4) and BUN were not significantly related to survival.

### Multivariate analyses

Three Cox proportional hazards regression models were developed (Table 4) using 556 patients (520 deaths) with complete data (excluding patients with missing data) based on the assumption of (a) a linear relationship between continuous covariates and log hazard, (b) a step functional dichotomisation of continuous covariates and (c) a nonlinear transformation of continuous covariates. All three models included trial, sex, cancer stage (stratification factors at randomisation) and randomised treatment group.

The ‘linear’ model (Table 4, Model 1) identified five highly significant prognostic factors, namely albumin, alkaline phosphatase, LDH, WBC and metastases. The ‘categorical’ model (Table 4, Model 2) identified six highly significant prognostic factors, namely LDH, albumin, metastases, WBC, CA19-9 and bilirubin. Univariate analysis of the 10 continuous variables identified that nonlinear transformations were appropriate for 3 variables in their relationship with survival: bilirubin and LDH both as log transformations and CA19-9 as a second-degree FP transformation (CA19-9^0.5^+(CA19-9^0.5^ × log(CA19-9))). The seven remaining continuous covariates were analysed assuming a linear relationship with log hazard, as in Model 1. The ‘transformed’ model (Table 4, Model 3) identified eight prognostic factors. Five factors were highly significant with *P*<0.01, namely albumin, CA19-9, LDH, alkaline phosphatase and WBC with AST, BUN and metastases being more borderline in the model (*P*=0.023, 0.026 and 0.047, respectively).

**Figure 2:**
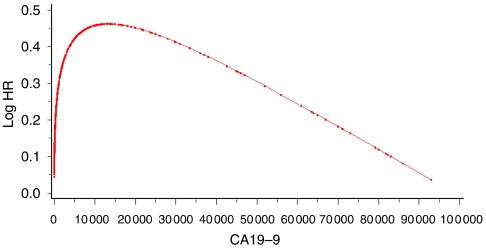
Estimated functional form for CA19-9. Dots indicate actual data values.

**Figure 3:**
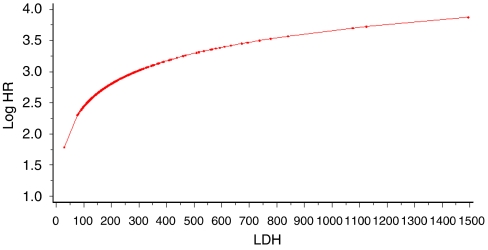
Estimated functional form for LDH. Dots indicate actual data values.

Nonlinear transformations were appropriate for two variables, LDH and CA19-9, and the estimated log hazard ratio functions are shown graphically in Figures 2 and 3. The second-degree FP function for CA19-9 estimates increasing risk up to an approximate CA19-9 value of 14 000 and then decreases with increasing CA19-9. The log function for LDH estimates increasing risk for increasing values of LDH.

### Model comparison

In all three models, albumin, LDH and WBC were highly statistically significant and influential prognostic factors. Metastases were also an important variable but its parameter estimate and overall significance were reduced in the ‘transformed’ model when continuous covariates were included in a more appropriate format. In both the ‘linear’ and ‘transformed’ models, alkaline phosphatase was also a highly significant and influential prognostic factor. CA19-9 was also a highly significant and influential prognostic factor in both the ‘categorical’ and ‘transformed’ models. This variation is largely explained by the nonlinear relation of CA19-9 to survival (Figure 2), which could explain why it was considered important when dichotomised but not when included as linear. When considered as a transformed second-degree FP, its significance was much greater. Bilirubin was selected as a highly significant factor in the ‘categorical’ model but was not included in either the ‘linear’ or ‘transformed’ models. AST and BUN were only selected as prognostic in the ‘transformed’ model.

### Model performance

The AIC statistic was smallest for the ‘transformed’ model (Table 4, Model 3), indicating a better fit to the data. Deviance residuals for this model were plotted against the linear predictor and were randomly scattered centred around a residual value of zero ranging between −3.86 and 3.33, which suggests the data have not been mis-modelled.

**Figure 4:**
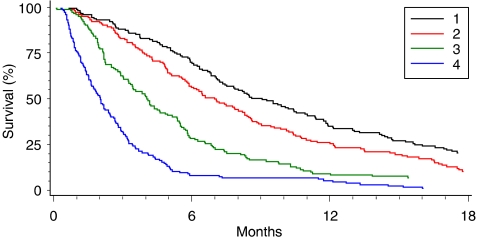
Survival by predictive group.

Patients were split into four groups based on quartiles of the distribution of linear predictor scores from the ‘transformed’ model. Kaplan–Meier survival estimates (Figure 4) show four distinct predictive groups with descending median survival estimates of 9.1 (95% CI: 7.4, 10.9), 7.0 (95% CI: 5.9, 8.3), 4.0 (95% CI: 3.4, 4.9) and 2.0 (95% CI: 1.6, 2.4) months. The hazard ratios for groups 2, 3 and 4 using predictive group 1 as the baseline were 1.35 (95% CI: 1.09, 1.66), 2.08 (95% CI: 1.64, 2.64) and 4.21 (95% CI: 3.11, 5.68), respectively.

When assessing model validity, the *R*^2^ measure of model fit was estimated as 0.30. The bootstrap resampled estimate of *R*^2^ of 0.26 described model optimism (overfitting) under 5% and gives an improved estimate of model accuracy.

Multiple imputation allowed all 653 patients to be included in the modelling process and confirmed all the variables included in the ‘transformed’ model with increased significance for metastases (*P*=0.001), and the model also included nodal status (*P*=0.016) that had been excluded from all models prior to imputation, suggesting a strong link with other variables already in the model.

## DISCUSSION

Large, prospective, phase III randomised controlled trials aim to provide robust statistical evidence for new treatment combinations. Stratification is important to control for known important variability in the data. Generally, patients with pancreatic cancer are not clinically separated into prognostic groups, with the exception of surgical status, before consideration for treatment. This study investigated potentially important baseline prognostic factors for survival as possible stratification variables for randomisation and analysis. Data from 653 patients included in two international randomised controlled trials in advanced pancreatic cancer (Bramhall *et al*, 2001, 2002) were analysed investigating multiple clinical, histological, biochemical and demographic variables in the form of both binary and continuous measurements. Valid statistical analyses are necessary to make best use of the data and optimise clinical results. As such, a multivariable approach was used to account for the functional form of the relationship between continuous prognostic variable factors and survival. Misspecification of functional form may lead to inappropriate conclusions but has not been previously investigated in pancreatic cancer studies. Continuous variables are often simplified by assuming a linear relationship between predictor and log hazard, that is the log risk increases or decreases linearly as the value of the factor increases, which may not be appropriate. Dichotomisation of continuous data is common but is problematic and unnecessary. As the variability in outcome within groups is ignored by categorisation, the variability between groups may be significantly underestimated as patients close to the cut point are analysed as being very different rather than being very similar, resulting in a serious reduction of statistical power to detect relationships between predictors and outcome, residual confounding and serious bias (Altman and Royston, 2006; Royston *et al*, 2006a). Regression using FPs of continuous covariates has been used in data from breast cancer (Sauerbrei *et al*, 1999) and metastatic renal carcinoma (Royston *et al*, 2006b) trials. Our study supported these in showing that this approach allowed important additional prognostic information to be extracted with less sophisticated approaches missed. FPs provide a flexible, parametric approach for modelling nonlinear relationships, making full use of the information available within each variable and as such can provide a clearer insight into the nature of the underlying relationship (Royston and Altman, 1994; Altman and Lyman, 1998).

Pancreatic ductal adenocarcinoma is the fifth most common cause of death from cancer in the Western world (Bramhall *et al*, 1995; Parkin *et al*, 2001; Jemal *et al*, 2003). It is particularly difficult to treat because of its remote location, late presentation and resistance to conventional chemotherapy. Long-term survival remains poor with a 5-year survival rate between 0.4 and 4% (Bramhall *et al*, 1995; Jemal *et al*, 2003). Resection is associated with improved survival but this is only possible in approximately 10% of patients (Sener *et al*, 1999; Alexakis *et al*, 2004). Although significant improvements in surgical outcome have been obtained with increasing specialisation (Neoptolemos *et al*, 1997; Birkmeyer *et al*, 1999), further benefits are anticipated by identifying high-risk groups. A validated prognostic index would identify subgroups of patients for specific treatments and predict survival, but there is no tool in routine use. Also, many possible prognostic factors have been identified in advanced pancreatic cancer (Table 1), most derived from retrospective studies based on small numbers of patients resulting in analyses that may be underpowered. A total of 34 possible factors were identified from 36 studies (4 randomised controlled trials; Johnson *et al*, 2001; Berlin *et al*, 2002; Ducreux *et al*, 2002; Maisey *et al*, 2002) and 32 consecutive series (Friedman and van den Eeden, 1993; Yasue *et al*, 1994; Falconer *et al*, 1995; Lundin *et al*, 1995; Ishii *et al*, 1996; Rothenberg *et al*, 1996; Shibamoto *et al*, 1996; Cubiella *et al*, 1999; Storniolo *et al*, 1999; Halm *et al*, 2000; Terwee *et al*, 2000; Trigui *et al*, 2000; Ueno *et al*, 2000; Ikeda *et al*, 2001; Ridwelski *et al*, 2001; Tas *et al*, 2001; Tsuruta *et al*, 2001; Saad *et al*, 2002; Bachmann *et al*, 2003; Engelken *et al*, 2003; Fujino *et al*, 2003; Karayiannakis *et al*, 2003; Micke *et al*, 2003; Ohigashi *et al*, 2003; Paillaud *et al*, 2003; Stemmler *et al*, 2003; Talar-Wojnarowska *et al*, 2003; Ziske *et al*, 2003; Gupta *et al*, 2004; Kuhlmann *et al*, 2004; Watanabe *et al*, 2004; Ni *et al*, 2005) of which 15 had <100 patients (Yasue *et al*, 1994; Ishii *et al*, 1996; Rothenberg *et al*, 1996; Halm *et al*, 2000; Ikeda *et al*, 2001; Tsuruta *et al*, 2001; Saad *et al*, 2002; Karayiannakis *et al*, 2003; Micke *et al*, 2003; Ohigashi *et al*, 2003; Paillaud *et al*, 2003; Stemmler *et al*, 2003; Talar-Wojnarowska *et al*, 2003; Ziske *et al*, 2003; Gupta *et al*, 2004), including demographic, clinical (including, performance status, weight loss and treatment), surgical (including, palliative procedures, site and stage of disease) and laboratory (including, CA19-9, LDH, alkaline phosphatase and albumin). Further concerns include the inadequate use of statistical methods, model comparison when different factors are being investigated and the differing format of factors across studies.

We developed three prognostic models, two based on standard assumptions of log linear or step functional relationships with survival and a novel approach based on nonlinear relationships using more complex FP transformations. The model based on transformed covariates (Table 4, Model 3) was the best-fitting model, better utilising the information within nonlinear covariates. This model confirmed five previously reported prognostic factors, namely albumin, CA19-9, alkaline phosphatase, LDH and metastases; and also identified three additional possible prognostic factors not previously reported: WBC, AST and BUN. Nonlinear transformations were appropriate for two variables indicating strong nonlinear effects on survival: CA19-9 as a second-degree FP and LDH under a log transformation. Importantly, the effect of CA19-9 was not apparent in the ‘linear’ model, the effect of alkaline phosphatase was not apparent in the ‘categorical’ model and the effects of AST and BUN were not apparent in either the ‘linear’ or ‘categorical’ models, indicating how the significant effect of these variables may go unrecognised due to simplistic assumptions made in statistical modelling.

Shrinkage represents the degree to which a plot of predicted and observed values is flattened from the 45° line attributable to overfitting. Overfitting leads to inflated estimates of model fit and is a potentially important source of bias in prognostic models. Overfitting may be minimised through sensible model selection, which for survival models implies avoiding attempting to fit models with more than 1 candidate variable (degree of freedom) for each 10 events of interest (e.g., death) included in the analysis. The degree to which overfitting is present in the fitted model may be estimated either directly through validation in an external data set or through a bootstrap process. In practise, it is rare for an external data set to be available, and if data are scarce, it becomes attractive to use all available data to derive the prognostic model. Thus, bootstrap resampling approaches may become the model validation methods of choice. In our model, bootstrap resampling methods (Harrell *et al*, 1996) suggested minimal optimism. As shrinking estimators will not increase the real discrimination of the model, and the degree of overfitting estimated for the model is minimal, rescaling the model estimates appears neither helpful nor necessary.

A model based on multiple imputation methods (Rubin, 1987; Schafer, 1997) to control for missing covariate data selected an additional variable nodal status as prognostic (*P*=0.016), which had been excluded from all models prior to imputation. The true importance of this variable requires further investigation, suggesting a strong link with other variables already in the model. All prognostic models ideally require external validation to determine the generality across different data sets, and our results may be seen as provisional until replicated on independent data. Performance status and tumour size at randomisation are well-documented factors (Table 1) but unfortunately were not available in this data set and should be included in any external validation.

This research was based on data from two large, phase III randomised controlled trials representative of patients with advanced pancreatic cancer with a high event rate, long follow-up and an overall 1 year survival rate of 17% (Bramhall *et al*, 2001, 2002). Analyses were based on a multivariable approach and utilised the information contained within continuous variables appropriately. The functional form of the relationship between continuous covariates and survival should always be assessed when investigating potential prognostic value. Models were based on information readily available in clinic and once validated should have the ability to aid decision-making by identifying patients with borderline disease for surgery and patients for inclusion into clinical trials or off-study treatment, especially since a greater number of palliative and more toxic treatments are becoming available and being trialed in this disease.

## Figures and Tables

**Table 1:** Literature review

**Type (number) of studies**	**Number of patients per study**	**Prognostic factors reported (frequency of reporting)**			**U or MV analysis**
Randomised controlled trial (*n*=4)^3–6^	207–322	Surgical	Metastases	(3)	4 MV
			Tumour location	(1)	
		Clinical	Performance status	(3)	
			Treatment	(1)	
		Laboratory	Alkaline phosphatase	(1)	
					
Consecutive series >500 patients (*n*=3)^7–9^	782–2380	Surgical	Metastases	(1)	2 MV
			Stage of disease	(1)	1 U
			Operation	(1)	
		Clinical	Performance status	(1)	
			Diabetes	(1)	
			Pain	(1)	
			Appetite/weight	(1)	
			Jaundice	(1)	
			Treatment	(1)	
		Laboratory	Albumin	(1)	
		Demographic	Age	(1)	
			Specialist centre	(1)	
Consecutive series 100–500 patients (*n*=14)^10–23^	102–450	Surgical	Metastases	(4)	13 MV
			Stage of disease	(2)	1 U
			Tumour location	(1)	
			Operation	(2)	
			Tumour size	(1)	
			Duodenal invasion	(1)	
			Peridissemination	(1)	
			Ascites	(1)	
		Clinical	Performance status	(2)	
			Diabetes	(1)	
			Pain	(1)	
			Appetite/weight	(3)	
			Symptom onset	(1)	
			Treatment	(2)	
		Laboratory	CA242	(2)	
			CA19-9	(2)	
			Leukocytes	(1)	
			Gamma GT	(1)	
			Albumin	(1)	
			LDH	(1)	
			CRP	(3)	
			Iron	(1)	
		Demographic	Age	(1)	
					
Consecutive series	28–95	Surgical	Metastases	(1)	8 MV
<100 patients (*n*=15)^24–38^			Stage of disease	(1)	7 U
			Grade of disease	(1)	
			Nodal status	(1)	
			Operation	(1)	
			Tumour size	(2)	
			Fibrosis	(1)	
		Clinical	Performance status Inflammation	(4)	
			Appetite/weight	(1)	
			Treatment	(1)	
		Laboratory	CA19-9	(1)	
			VEGF	(7)	
			CEA	(1)	
			Phase angle BIA	(1)	
			SCA	(1)	
				(1)	

MV=multivariate; U=univariate.

**Table 2:** Patient characteristics by trial

**Variable**	**BB128^39^ *N*=414 (63%)**	**BB193^40^ *N*=239 (37%)**	**Total *N*=653 (100%)**
**Demographics**			
*Age at entry (years)* a			
Median	63	62	63
Range	29–89	32–85	29–89
			
*Ethnic race*			
White	364 (88%)	226 (95%)	590 (90%)
Black	27 (6%)	8 (3%)	35 (6%)
Oriental	7 (2%)	0	7 (1%)
Other	15 (4%)	5 (2%)	20 (3%)
Missing	1	0	1
			
*Sex*			
Male	228 (55%)	140 (59%)	368 (56%)
Female	186 (45%)	99 (41%)	285 (44%)
			
*Treatment*			
Gemcitabine	103 (25%)	119 (50%)	222 (34%)
Marimistat	311 (75%)	120 (50%)	431 (66%)
			
**Tumour information**			
*Cancer stage*			
I	19 (4%)	13 (5%)	32 (5%)
II	45 (11%)	27 (11%)	72 (11%)
III	76 (19%)	28 (12%)	104 (16%)
IV	268 (66%)	171 (72%)	439 (68%)
Missing	6	0	6
			
*Distant metastases*			
M0	129 (31%)	65 (27%)	194 (30%)
M1	265 (64%)	171 (72%)	436 (67%)
Missing	20 (5%)	3 (1%)	23 (3%)
			
*Regional lymph nodes*			
N0	153 (37%)	90 (38%)	243 (37%)
N1	164 (40%)	87 (36%)	251 (39%)
Missing	97 (23%)	62 (26%)	159 (24%)
			
*Primary tumour T stage*			
T0	5 (1%)	3 (1.5%)	8 (1.5%)
T1	114 (30%)	44 (20%)	158 (26%)
T2	90 (24%)	54 (25%)	144 (24%)
T3	167 (44%)	113 (53%)	280 (47%)
T4	6 (1%)	1 (0.5%)	7 (1.5%)
Missing	32	24	56
			
**Serum chemistry and haematology**			
**Laboratory variables**	**Median (range), missing**	**Median (range), missing**	**Median (range), missing**
AST (SGOT)^a^	24 (6–365), 17	26 (9–538), 12	25 (6–538), 29
Total bilirubin^a^	13.7 (3.4–277.0), 16	13.7 (3.0–135.1), 8	13.7 (3.0–277.0), 24
Alkaline phosphatase^a^	136 (36–1660), 16	157 (35–2064), 8	140 (35–2064), 24
Albumin^a^	38 (22–47), 17	38 (24–47), 8	38 (22–47), 25
LDH^a^	163 (77–1074), 21	169 (29–1495), 11	164 (29–1495), 32
BUN^a^	9.2 (2.9–34.3), 17	9.3 (4.3–27.9), 16	9.3 (2.9–34.3), 33
CA19/9^a^	686 (5–1 000 000), 17	800 (8–1 000 000), 30	710 (5–1 000 000), 47
Haemoglobin^a^	12.5 (5.5–16.1), 28	12.4 (8.3–19.1), 13	12.4 (5.5–19.1), 41
WBC^a^	7.6 (2.3–31.6), 28	8.3 (2.4–23.7), 13	7.9 (2.3–31.6), 41
			
**Outcome**			
*Event indicator*			
Alive	22 (5%)	19 (8%)	41 (6%)
Dead	392 (95%)	220 (92%)	612 (94%)
			
*Follow-up of alive (months)* a			
Median	20.1	19.4	20.7
Range	0.9–24.6	1.9–23.3	0.9–24.6

a Continuous measurements.

**Table 3:** Univariate log-rank analyses

	**Patients**	**Deaths**	**12-month survival (%)**	**Median survival (95% CI)**	***χ*^2^_LR_, p (*χ*^2^_W_, p)**	**HR (95% CI)**
Overall survival	653	612	17	4.7 (4.2, 5.1)	—	—
						
*Trial*						
BB128	414	392	17	4.2 (3.6, 4.8)	2.28, *P*=0.13	1.0
BB193	239	220	18	5.4 (4.8, 6.0)		0.88 (0.75, 1.04)
						
**Demographics**						
*Age group (years)*						
<=63	343	320	21	5.1 (4.3, 5.8)	4.42, *P*=0.036	1.0
>63	310	292	13	4.3 (3.5, 4.9)	(7.55, *P*=0.006)	1.18 (1.01, 1.39)
						
*Ethnic group*						
White	590	554	18	4.6 (4.1, 5.1)	0.20, *P*=0.65	1.0
Other	62	57	12	5.2 (3.5, 6.0)		1.06 (0.80, 1.41)
						
*Sex*						
Female	285	267	18	4.9 (4.2, 5.8)	0.73, *P*=0.39	1.0
Male	368	345	17	4.5 (3.9, 5.1)		1.07 (0.91, 1.26)
						
*Treatment*						
Gemcitabine	222	204	18	5.5 (4.7, 5.9)	2.79, *P*=0.095	1.0
Marimistat	431	408	17	4.2 (3.5, 4.9)		1.15 (0.98, 1.36)
						
**Tumour information**						
*Cancer stage*						
Early (I/II)	104	92	26	6.8 (5.7, 8.2)	14.72, *P*<0.001	1.0
Late (III/IV)	543	514	16	4.1 (3.5, 4.7)		1.53 (1.26, 1.86)
						
*Metastases*						
M0	194	176	30	6.8 (5.9, 8.4)	35.47, *P*<0.001	1.0
M1	436	414	12	3.5 (3.2, 4.0)		1.69 (1.43, 1.99)
Missing	23	22	17	5.5 (4.9, 7.5)		1.35 (0.88, 2.09)
						
*Lymph nodes*						
N0	243	226	20	5.5 (4.8, 6.0)	6.73, *P*=0.035	1.0
N1	251	240	18	4.5 (3.5, 5.4)		1.19 (1.00, 1.43)
Missing	159	146	11	3.8 (3.2, 4.9)		1.29 (1.04, 1.59)
						
*Tumour stage*						
Early (0/1/2)	310	291	16	4.3 (3.7, 4.9)	1.63, *P*=0.44	1.0
Late (3/4)	287	268	18	4.9 (4.2, 5.8)		0.91 (0.77, 1.08)
Missing	56	53	18	5.8 (3.5, 7.9)		0.87 (0.66, 1.16)
						
**Serum chemistry and haematology**						
*AST (SGOT)*						
Normal	538	499	19	5.1 (4.6, 5.7)	14.17, *P*<0.001	1.0
Abnormal	86	84	12	2.8 (2.2, 3.9)	(5.99, *P*=0.014)	1.55 (1.18, 2.04)
						
*Total bilirubin*						
Normal	464	429	20	5.1 (4.7, 5.7)	9.32, *P*=0.002	1.0
Abnormal	165	159	11	3.8 (3.3, 4.4)	(6.27, *P*=0.012)	1.32 (1.09, 1.61)
						
*Alkaline phosphatase*						
Normal	442	411	20	5.5 (5.0, 6.1)	20.20, *P*<0.001	1.0
Abnormal	187	177	13	3.1 (2.6, 3.5)	(56.05, *P*<0.001)	1.49 (1.23, 1.81)
						
*Albumin*						
Normal	583	544	19	5.1 (4.6, 5.6)	31.37, *P*<0.001	1.0
Abnormal	45	43	7	1.5 (1.0, 2.7)	(74.34, *P*<0.001)	2.36 (1.49, 3.72)
						
*LDH*						
Normal	543	505	20	5.2 (4.8, 5.8)	37.05, *P*<0.001	1.0
Abnormal	78	75	5	2.1 (1.5, 2.8)	(36.16, *P*<0.001)	2.08 (1.50, 2.88)
						
*BUN*						
Normal	407	382	20	5.1 (4.3, 5.7)	3.43, *P*=0.064	1.0
Abnormal	213	199	13	4.4 (3.5, 5.1)	(5.28, *P*=0.022)	1.17 (0.98, 1.40)
						
*CA19/9*						
Normal	98	86	28	6.3 (4.8, 8.0)	7.74, *P*=0.005	1.0
Abnormal	508	481	16	4.6 (4.0, 5.1)	(4.84, *P*=0.028)	1.38 (1.12, 1.70)
						
*Haemoglobin*						
Normal	79	77	8	3.7 (3.3, 5.1)	6.88, *P*=0.009	1.0
Abnormal	533	495	20	4.9 (4.4, 5.6)	(10.64, *P*=0.001)	0.73 (0.55, 0.95)
						
*WBC*						
Normal	483	446	21	5.5 (4.9, 5.9)	34.36, *P*<0.001	1.0
Abnormal	129	126	8	2.9 (2.4, 4.0)	(46.52, *P*<0.001)	1.78 (1.40, 2.26)

HR=hazard ratio; LR=log-rank statistic; W=Wald *χ*^2^ statistic.

**Table 4:** Cox proportional hazards regression models, *n*=556 patients, 520 deaths

	**Variable**	***χ* ^2^**	***P*-value**	**HR (95% CI)**
***(a) Model 1 – ‘Linear’ covariates***				
**Full model**				
*AIC*=*5558.4*				
Stratification factors	Trial	7.3	0.007	0.76 (0.63, 0.93)
	Cancer stage^a^	0.0001	0.99	1.00 (0.69, 1.44)
	Sex	3.8	0.050	0.82 (0.68, 1.00)
	TRT	2.6	0.11	1.17 (0.97, 1.42)
Independent factors	Age	3.0	0.081	1.01 (1.00, 1.02)
	Albumin	22.9	<0.001	0.94 (0.92, 0.96)
	Alkaline phosphatase	24.3	<0.001	1.00 (1.00, 1.00)
	AST	5.3	0.022	1.00 (0.99, 1.00)
	Bilirubin	0.7	0.40	1.00 (1.00, 1.01)
	BUN	2.3	0.13	1.02 (0.99, 1.05)
	CA19/9	0.9	0.34	1.00 (1.00, 1.00)
	Ethnic	0.0009	0.98	1.01 (0.74, 1.36)
	Haemoglobin	0.01	0.91	1.00 (0.94, 1.08)
	LDH	19.3	<0.001	1.00 (1.00, 1.00)
	METS^b^	8.5	0.004	1.50 (1.14, 1.96)
	Nodes^b^	0.4	0.51	1.08 (0.86, 1.37)
	Tumour stage^b^	0.03	0.86	1.02 (0.84, 1.23)
	WBC	10.2	0.001	1.04 (1.02, 1.07)
				
**Final model**				
*AIC*=*5557.1*				
Stratification factors	Trial	8.1	0.005	0.76 (0.63, 0.92)
	Cancer stage^a^	0.008	0.93	1.01 (0.74, 1.38)
	Sex	3.8	0.051	0.84 (0.70, 1.00)
	TRT	3.2	0.073	1.19 (0.98, 1.44)
Independent factors	Albumin	41.0	<0.001	0.94 (0.92, 0.96)
	LDH	13.3	<0.001	1.00 (1.00, 1.00)
	METS^b^	10.5	0.001	1.50 (1.17, 1.92)
	WBC	11.7	<0.001	1.04 (1.02, 1.07)
	**Alkaline phosphatase**	**21.1**	**<0.001**	**1.00 (1.00, 1.00)**
				
***(b) Model 2 – ‘Categorical’ covariates***				
**Full model**				
*AIC*=*5582.3*				
Stratification factors	Trial	5.8	0.016	0.79 (0.65, 0.96)
	Cancer stage^a^	0.0001	0.99	1.00 (0.70, 1.44)
	Sex	2.1	0.14	0.87 (0.72, 1.05)
	TRT	3.3	0.07	1.19 (0.99, 1.44)
Independent factors	Age	2.2	0.13	1.15 (0.96, 1.39)
	Albumin	17.6	<0.001	2.08 (1.48, 2.93)
	Alkaline phosphatase	0.3	0.57	1.07 (0.85, 1.34)
	AST	1.5	0.22	1.20 (0.90, 1.59)
	Bilirubin	5.0	0.025	1.28 (1.03, 1.58)
	BUN	1.9	0.17	1.14 (0.94, 1.39)
	CA19/9	7.8	0.005	1.43 (1.11, 1.85)
	Ethnic	0.5	0.48	0.89 (0.66, 1.22)
	Haemoglobin	2.2	0.14	0.82 (0.62, 1.07)
	LDH	22.8	<0.001	2.07 (1.54, 2.79)
	METS^b^	13.3	<0.001	1.64 (1.26, 2.14)
	Nodes^b^	2.3	0.13	1.20 (0.95, 1.51)
	T stage^b^	0.06	0.80	0.98 (0.81, 1.18)
	WBC	9.2	0.002	1.42 (1.13, 1.78)
				
**Final model**				
*AIC*=*5583.2*				
Stratification factors	Trial	7.0	0.008	0.77 (0.64, 0.94)
	Cancer stage^a^	0.9	0.36	1.16 (0.85, 1.58)
	Sex	1.8	0.18	0.89 (0.74, 1.06)
	TRT	3.4	0.065	1.19 (0.99, 1.44)
Independent factors	Albumin	24.2	<0.001	2.30 (1.65, 3.21)
	LDH	25.1	<0.001	2.05 (1.55, 2.72)
	METS^b^	11.9	<0.001	1.54 (1.21, 1.97)
	WBC	10.3	0.001	1.44 (1.15, 1.79)
	**Bilirubin**	**8** **.4**	**0** **.004**	**1.34** **(1.10, 1.64)**
	**CA199**	**9** **.4**	**0** **.002**	**1.48** **(1.15, 1.89)**
***(c) Model 3–‘Transformed’ covariates***				
**Full model**				
*AIC*=*5509.6*				
Stratification factors	Trial	12.5	<0.001	0.70 (0.57, 0.85)
	Cancer stage^a^	0.2	0.68	0.93 (0.64, 1.33)
	Sex	4.3	0.038	0.82 (0.67, 0.99)
	TRT	3.0	0.082	1.19 (0.98, 1.44)
				
*Independent factors*				
Linear	Age	2.3	0.13	1.01 (1.00, 1.02)
Linear	Albumin	20.8	<0.001	0.94 (0.92, 0.97)
Linear	Alkaline phosphatase	11.3	<0.001	1.00 (1.00, 1.00)
Linear	AST	7.5	0.0062	1.00 (0.99, 1.00)
1st degree FP	Log (bilirubin)	3.0	0.083	1.16 (0.98, 1.37)
Linear	BUN	3.2	0.075	1.02 (1.00, 1.05)
2nd degree FP	CA199^0.5^	36.3	<0.001	1.03 (1.02, 1.03)
2nd degree FP	CA199^0.5^ × log(CA199)	30.9	<0.001	1.00 (1.00, 1.00)
Categorical	Ethnic	0.1	0.74	0.95 (0.70, 1.29)
Linear	Haemoglobin	0.3	0.61	0.98 (0.92, 1.05)
1st degree FP	Log (LDH)	12.3	<0.001	1.72 (1.27, 2.32)
Categorical	METS^b^	6.1	0.014	1.41 (1.07, 1.85)
Categorical	Nodes^b^	1.4	0.24	1.15 (0.91, 1.46)
Categorical	Tumour stage^b^	0.3	0.60	1.05 (0.87, 1.28)
Linear	WBC	10.5	0.0012	1.05 (1.02, 1.07)
				
**Final model**				
*AIC*=*5510.2*				
Stratification factors	Trial	15.1	<0.001	0.68 (0.56, 0.83)
	Cancer stage^a^	0.001	0.97	1.0 (0.73, 1.36)
	Sex	3.5	0.061	0.84 (0.70, 1.01)
	TRT	3.16	0.075	1.19 (0.98, 1.44)
				
*Independent factors*				
Linear	Albumin	41.4	<0.001	0.94 (0.92, 0.95)
1st degree FP	Log (LDH)	12.8	<0.001	1.70 (1.27, 2.27)
Categorical	METS^b^	4.0	0.047	1.29 (1.00, 1.66)
Linear	WBC	10.0	0.002	1.04 (1.02, 1.07)
**Linear**	**Alkaline phosphatase**	**14** **.6**	**<0** **.001**	**1.00** **(1.00, 1.00)**
**Linear**	**AST**	**5** **.2**	**0** **.023**	**1.00** **(0.99, 1.00)**
**Linear**	**BUN**	**5** **.0**	**0** **.026**	**1.03** **(1.00, 1.06)**
**2nd degree FP**	**CA199** ^**0.5**^	**33** **.3**	**<0** **.001**	**1.02** **(1.02, 1.03)**
**2nd degree FP**	**CA199^0.5^ × log(CA199)**	**28.4**	**<0.001**	**1.00 (1.00, 1.00)**

FP=fractional polynomial; HR=hazard ratio.

a Stage (I/II *vs* III/IV).

b Metastases (negative *vs* positive), Nodes (negative *vs* positive), Tumour stage (I/II *vs* III/IV): missing data included in analysis as a separate ‘dummy’ variable using lower level as the reference level.
